# A structural equation model of the relationship between muscle strength, balance performance, walking endurance and community integration in stroke survivors

**DOI:** 10.1371/journal.pone.0185807

**Published:** 2017-10-19

**Authors:** P. W. H. Kwong, S. S. M. Ng, R. C. K. Chung, G. Y. F. Ng

**Affiliations:** Department of Rehabilitation Sciences, The Hong Kong Polytechnic University, Hong Kong, China; The Ohio State University, UNITED STATES

## Abstract

**Purpose:**

To use structural equation modelling (SEM) to determine (1) the direct and indirect associations of strength of paretic lower limb muscles with the level of community integration, and (2) the direct association of walking endurance and balance performance with the level of community integration in community-dwelling stroke survivors.

**Materials and methods:**

In this cross-sectional study of 105 stroke survivors, the Subjective Index of Physical and Social Outcome (SIPSO) was used to measure the level of community integration. Lower-limb strength measures included isometric paretic ankle strength and isokinetic paretic knee peak torque. The Berg Balance Scale (BBS) and the 6-minute walk test (6MWT) were used to evaluate balance performance and walking endurance, respectively.

**Results:**

SEM revealed that the distance walked on the 6MWT had the strongest direct association with the SIPSO score (*β* = 0.41, *p* <0.001). An increase of one standard deviation in the 6MWT distance resulted in an increase of 0.41 standard deviations in the SIPSO score. Moreover, dorsiflexion strength (*β* = 0.18, *p =* 0.044) and the BBS score (*β* = 0.21, *p* = 0.021) had direct associations with the SIPSO score.

**Conclusions:**

The results of the proposed model suggest that rehabilitation training of community-dwelling stroke survivors could focus on walking endurance, balance performance and dorsiflexor muscle strengthening if the aim is to augment the level of community integration.

## Introduction

Stroke is a major cause of motor impairment and disability worldwide [1]. The residual physical and cognitive deficiencies after stroke can reduce both the quality and the quantity of the survivor’s social participation and interaction [2, 3]. Impaired physical and cognitive functions, changes in social roles, the inability to work and poor adaptability to the physical environment can hinder the process of community integration for stroke survivors, which in turn can lead to social isolation [4]. One study reported that only 11% of community-dwelling stroke survivors were completely satisfied with their level of community integration [5].

Promoting successful community integration after a stroke is one of the ultimate goals of rehabilitation. Identification of predictors for community integration can help researchers and clinicians develop new treatment strategies and enhance the process of community integration after hospital discharge. Obembe et al. [6] reported that age, the geriatric depression scale (GDS) score and the motor assessment scale score were significant predictors of stoke survivors’ scores on the return to normal living index (RNLI; *R*^*2*^ = 41.0%). Chau et al. [7] showed that GDS score (*β* = −0.41), gender (*β* = 0.11), age (*β* = −0.13) and living in a residential care institute (*β* = −0.11) significantly predicted the London handicap scale (LHS) score (*R*^*2*^ = 71.0%). Physical functioning, such as balance performance measured with the Berg Balance Scale (BBS), was an independent predictor of the RNLI score (*β* = 0.36) [5]. Although walking endurance measured with the 6-minute walk test (6MWT) predicted the level of community integration as measured by the RNLI, neither the strength of the association nor the *R*^*2*^ value of the model was reported [8].

Muscle weakness is a common physical impairment following a stroke [9]. The concentric paretic knee extension torque measured with an isokinetic dynamometer has been shown to be significantly correlated with self-paced sit-to-stand duration (*r* = −0.72) [10], comfortable walking speed (*r* = 0.61) [11], maximum walking speed (*r* = 0.65 to 0.85) [11, 12] and speed of walking up stairs (*r* = −0.58) [11]. Isometric paretic ankle dorsiflexion and plantarflexion strength were significantly correlated with the comfortable walking speed (*r* = 0.77 and 0.83, respectively) and maximum walking distance (*r* = 0.68 and 0.76, respectively), which measures the distance the subject is able to walk before stopping due to fatigue [13]. Moreover, paretic ankle dorsiflexion strength was found to be an independent predictor of the timed up and go test completion time (*R*^*2*^ = 27.5%)[14] and the 6MWT distance (*R*^*2*^ = 48.4%) [15]. The strength of the paretic lower limb muscles was a strong predictor of functional performance [14, 15], including walking endurance [8] and balance [5], which were identified as independent predictors of the level of community integration. Thus, it is plausible that muscle strength has a strong influence on the level of community integration, as mediated by the level of functional performance. However, no studies have evaluated the relationship between paretic lower-limb muscle strength and level of community integration in stroke survivors.

In accordance with the International Classification of Functioning, Disability and Health model [16], we hypothesised that paretic lower-limb muscle strength would have a direct association with balance performance and walking endurance and thus be indirectly associated with the level of community integration in stroke survivors. Therefore, the aim of this study was to determine (1) the direct and indirect associations of the muscle strength of the paretic lower limb with the level of community integration, and (2) the direct associations of balance performance and walking endurance with the level of community integration in community-dwelling stroke survivors.

## Methods

### Procedure

Ethical approval has been obtained from The Hong Kong Polytechnic University. The study was conducted in accordance with the Declaration of Helsinki ethical principles for human experimentation, and written consent was obtained from all participants before the assessment. All of the assessments were conducted by the same physical therapist in one session.

### Participants

The subjects were recruited via posters at local self-help group centres for stroke survivors. Subjects were included if they (1) were between 50 and 85 years of age; (2) had a stroke at least 1 year earlier; (3) received a diagnosis of ischaemic brain injury or intracerebral haemorrhage by magnetic resonance imaging or computed tomography; (4) were able to walk independently for 10 m with or without a walking aid; (5) were able to follow instructions and give informed consent; and (6) scored greater than 6 of 10 on the abbreviated mental test (AMT), which indicates normal cognitive function [17]. Subjects were excluded if they (1) had any medical, cardiovascular or orthopaedic conditions that would hinder assessment or (2) were involved in drug studies or other clinical trials.

### Outcome measures

#### Level of community integration

The Subjective Index of Physical and Social Outcome (SIPSO) is a 10-item questionnaire specifically designed to assess the level of community integration in stroke survivors and their satisfaction with their functioning status [2, 18]. Each item is rated on a five-point ordinal scale (0 to 4). SIPSO has demonstrated excellent test–retest reliability with an intraclass correlation coefficient (ICC) of 0.87 to 0.91 [19, 20]. The total SIPSO score has been shown to be significantly correlated with the Barthel index, Frenchay activities index, Wakefield depression inventory and Nottingham health profile in chronic stroke survivors (Spearman’s ρ: 0.67 to 0.80) [19]. The Chinese version of SIPSO was used in this study [20].

#### Isometric ankle muscle strength

The maximum isometric dorsiflexion and plantarflexion strength of the paretic ankle were measured with a Nicholas handheld dynamometer (Lafayette Instrument Company, Lafayette, IN) in a supine lying position. Good to excellent reliability (ICC range, 0.84 to 0.99) has been reported when using the handheld dynamometer to assess lower-limb muscle strength in chronic stroke survivors [21]. At least 1 minute of rest was given between each set of isometric contractions of the ankle muscles to avoid muscle fatigue. The handheld dynamometer was placed on the dorsal and plantar surfaces across the first to fifth metatarsal-phalangeal joints with the ankle at 0 degrees of dorsiflexion.

#### Isokinetic knee muscle strength

The peak concentric isokinetic torque of the paretic knee extensor and flexor were measured at 90°/s in the sitting position with an isokinetic machine (Cybex 6000 dynamometer, Cybex, Henley, USA). An angular velocity of 90°/s was selected because good test–retest reliability was reported when measuring the peak isokinetic torque of the knee extensor (ICC, 0.81) in chronic stroke survivors using an angular velocity of 90°/s [22], but not with a lower angular velocity of 30°/s (ICC, 0.42). Another study reported that most stroke survivors were able to achieve an angular velocity of 90°/s and that only 1.3% of the trials were discarded due to a mismatch between the actual movement speed and the criterion speed. In contrast, 5.2% of the trials in which an angular velocity of 120°/s was used were discarded due to mismatches [23].

#### Balance performance

The Berg Balance Scale (BBS) was used to measure functional balance performance. The scale consists of 14 items, each rating a participant’s ability to maintain stability while completing a specified functional task on a 5-point (0 to 4) scale [24]. The BBS has excellent test–retest reliability (ICC, 0.95 to 0.98) [25] and inter-rater reliability (ICC, 0.95) [26] in assessing stroke survivors. Participants could wear an ankle/foot orthosis but were not allowed to use a walking aid in this assessment.

#### Walking endurance

The distance covered in a 6MWT [27] was used to assess walking endurance. The participants were instructed to walk as far as possible within 6 minutes. Excellent test–retest reliability for the walking distance covered (ICC, 0.99) was demonstrated in a previous study of chronic stroke survivors [27]. The assessment protocols followed those recommended by the American Thoracic Society [28], except that the walkway length was 15 m due to the space limitations of our laboratory. Participants were instructed to walk back and forth on the 15 m walkway, turning around as they reached either end of the walkway. The participants were allowed to wear ankle/foot orthosis and use a walking aid as necessary.

### Statistical analysis

Statistical analyses were conducted with SPSS (version 22.0. Armonk, NY: IBM Corp.) in conjunction with AMOS (version 23.0, Chicago: IBM SPSS). A *p* value of 0.05 or less was considered to indicate statistical significance. The participants’ demographic characteristics are summarised with descriptive statistics. The level of depression symptoms was quantified using the geriatric depression scale (GDS) [29].

The statistical analyses of the relationship between the SIPSO and the functional outcomes were conducted in two stages. In the first stage, partial correlation analysis was used to evaluate the strength of associations between the SIPSO score and other physical outcomes after controlling for the effects of age, gender and the GDS score. The variables that demonstrated a significant correlation with the SIPSO score were further analysed with multiple linear regression with a stepwise approach. To avoid the inflation of type 1 errors, a Bonferroni correction was applied in the partial correlation analyses. Regression analysis was used to identify the set of variables that could independently explain part of the variance of the SIPSO score. Similarly, paretic lower-limb muscle strength demonstrated a significant partial correlation with functional performance and was further analysed by multiple linear regression, with functional performance as the dependent variable. The variance inflation factor was used to assess the degree of multicollinearity in the regression models. A variance inflation factor greater than 10 indicated that a particular predictor demonstrated a strong linear relationship with other predictors in the model [30].

The second stage of the statistical analysis involved the development of a hypothesised model based on the results of the multiple regression analysis. We hypothesised that muscle strength would directly influence functional performance and that functional performance would directly influence the SIPSO scores. The variables that were found to be significant predictors in the multiple regression analysis were selected to construct the model. Structural equation modelling (SEM) is a multivariate statistical analysis technique that estimates the strength of the direct and indirect relationships between sets of variables. We used SEM to estimate the strength and significance of the hypothesised causal connections between the paretic lower-limb muscle strength, balance performance, walking endurance and SIPSO score. The SIPSO score and outcome measures at the functional level were considered to be endogenous variables. Paretic lower-limb muscle strength was considered to be an exogenous variable.

A path diagram was used to represent the interconnections of each variable in the model. In the path diagram, a single-headed arrow indicates a direct association, with the arrow head pointing to the dependent variable and its tail from the independent variable. A curved arrow indicates a correlation between two variables. An indirect association signifies that an independent variable could influence the dependent variable, mediated by another variable.

The model fit was assessed with the comparative fit index (CFI) and the standardised root mean square residual (SRMR) [31]. If either index showed a poor fit of the data to our hypothesised model, we would revise the entire model according to the recommendations given by the modification index, which estimates the effect on the chi-square statistic of adding an additional path to the model [32]. A modification index value larger than 3.84 for a specific path indicates that adding the path to the model could significantly improve the model fit [32]. The minimal sample size for the SEM was suggested to be at least 15 cases per predictor in the hypothesised model. Thus, our sample of 105 stroke survivors was sufficient to conduct SEM.

## Results

One-hundred and five community-dwelling stroke survivors (60% male) were recruited for this study. The mean time since stroke was 6.2 years, and the subjects’ mean age was 61.0 years. The majority were living with family or friends (87.6%), and only eight (7.6%) were still in the workforce (Table 1).

**Table 1:** Demographic characteristics of the participants and results of the physical assessments (n = 105).

Variable	Frequency (%)
Gender (male/female)	63 (60.0)/42 (40.0)
Side of hemiplegia (right/left)	57 (54.3)/48 (45.7)
Type of stroke (ischaemic/haemorrhagic/mixed)	59 (56.1)/43 (41.0)/3 (2.9)
Living with care giver (yes/no)	92 (87.6)/13 (12.4)
Working (yes/no)	8 (7.6)/97 (92.4)
Education level (primary or below/secondary/college or above)	31 (29.5)/60 (57.1)/14 (13.3)
Using walking aid (yes/no)	69 (65.8)/36 (34.2)
Using ankle foot orthosis (yes/no)	6 (5.7)/99 (94.3)
	Mean ± SD (range)
Age (y)	61.0 ± 6.9 (50.0 to 79.0)
Time since stroke (y)	6.2 ± 4.9 (1.0 to 24.0)
Paretic ankle dorsiflexion strength (kg)	8.5 ± 5.0 (0.6 to 23.7)
Paretic ankle plantarflexion strength (kg)	13.4 ± 5.6 (2.3 to 28.4)
Paretic knee extension torque (Nm)	23.7 ± 14.2 (4.0 to 85.0)
Paretic knee flexion torque (Nm)	7.53 ± 6.2 (0.0 to 26.0)
6MWT (m)	219.7 ± 84.4 (35.0 to 452.8)
	Median, IQR (range)
AMT	10, 1 (7 to 10)
BBS	48, 7 (17 to 56)
GDS	9, 8 (0 to 28)
SIPSO	28, 10 (10 to 40)

6MWT: 6-minute walk test; AMT: Abbreviated Mental Test; BBS: Berg Balance Scale; GDS: Geriatric Depression Score; IQR: interquartile range; SD: standard deviation; SIPSO: Subject Index of Physical and Social Outcome.

After controlling for the effects of age, gender and GDS score, isometric paretic ankle dorsiflexion strength, isokinetic paretic knee flexion torque, BBS scores and the distance covered in the 6MWT were found to demonstrate significant correlations with the SIPSO score. In addition, isometric paretic ankle dorsiflexion strength, isokinetic paretic knee extension torque and isokinetic paretic knee flexion torque showed significant correlations with BBS scores and distance covered in the 6MWT, but isometric paretic ankle plantarflexion strength did not show such a correlation (Table 2).

**Table 2:** Partial correlation coefficients (controlling for GDS score, gender and age) between SIPSO score, functional performance and muscle strength (n = 105).

	Correlation coefficient						
Variable	1	2	3	4	5	6	7
1. SIPSO	1						
2. Paretic dorsiflexion strength	0.447**	1					
3. Paretic plantarflexion strength	0.218	0.234	1				
4. Paretic knee extension torque	0.235	0.249	0.005	1			
5. Paretic knee flexion torque	0.287	0.310*	0.054	0.598**	1		
6. 6-minute walk test	0.545**	0.413**	0.092	0.401*	0.458**	1	
7. Berg Balance Scale	0.429**	0.402**	0.212	0.306*	0.249	0.464**	1

*p <0.05

**p <0.001

GDS: Geriatric Depression Score; SIPSO: Subjective Index of Physical and Social Outcome.

*p* value was adjusted with Bonferroni correction.

Multiple linear regression analyses showed that isometric ankle dorsiflexion strength, BBS score and distance covered in 6MWT were significant predictors of SIPSO score, with the whole model explaining 43.1% of the variance of the SIPSO score (Table 3). Isometric ankle dorsiflexion strength and isokinetic paretic knee flexion torque were significant predictors of the distance covered in the 6MWT (*R*^*2*^ = 32.5%). Isometric ankle dorsiflexion strength and isokinetic paretic knee extension torque were significant predictors of the BBS score (*R*^*2*^ = 25.0%). The variance inflation factors ranged from 1.07 to 1.55, indicating that some correlations exist between the predictors but that they may not be problematic.

**Table 3:** Hierarchical multiple regression analyses for the hypothesised model (n = 105).

Dependent variable	*R*^*2*^	Independent variable	Standardised coefficient *β*	*p*
SIPSO score	0.431	DF strength	0.17	0.048*
		6MWT	0.41	<0.001**
		BBS	0.21	0.029*
6MWT	0.325	DF strength	0.30	0.001*
		Knee F torque	0.39	<0.001**
BBS	0.250	DF strength	0.40	<0.001**
		Knee Ext torque	0.21	0.018

6MWT: 6-meter walk test; BBS: Berg Balance Score; DF: dorsiflexion; Ext: extension; F: flexion; SIPSO: Subjective Index of Physical and Social Outcome.

*p <0.05

**p <0.001

The hypothesised model was constructed based on the results of the hierarchical multiple regression analysis. The SEM supported the hypothesis that BBS score and the 6MWT distance would have a direct association with the SIPSO score. Isometric ankle dorsiflexion strength also had an indirect association with the SIPSO score, which was mediated by its influences on the BBS score and 6MWT distance.

**Fig 1:**
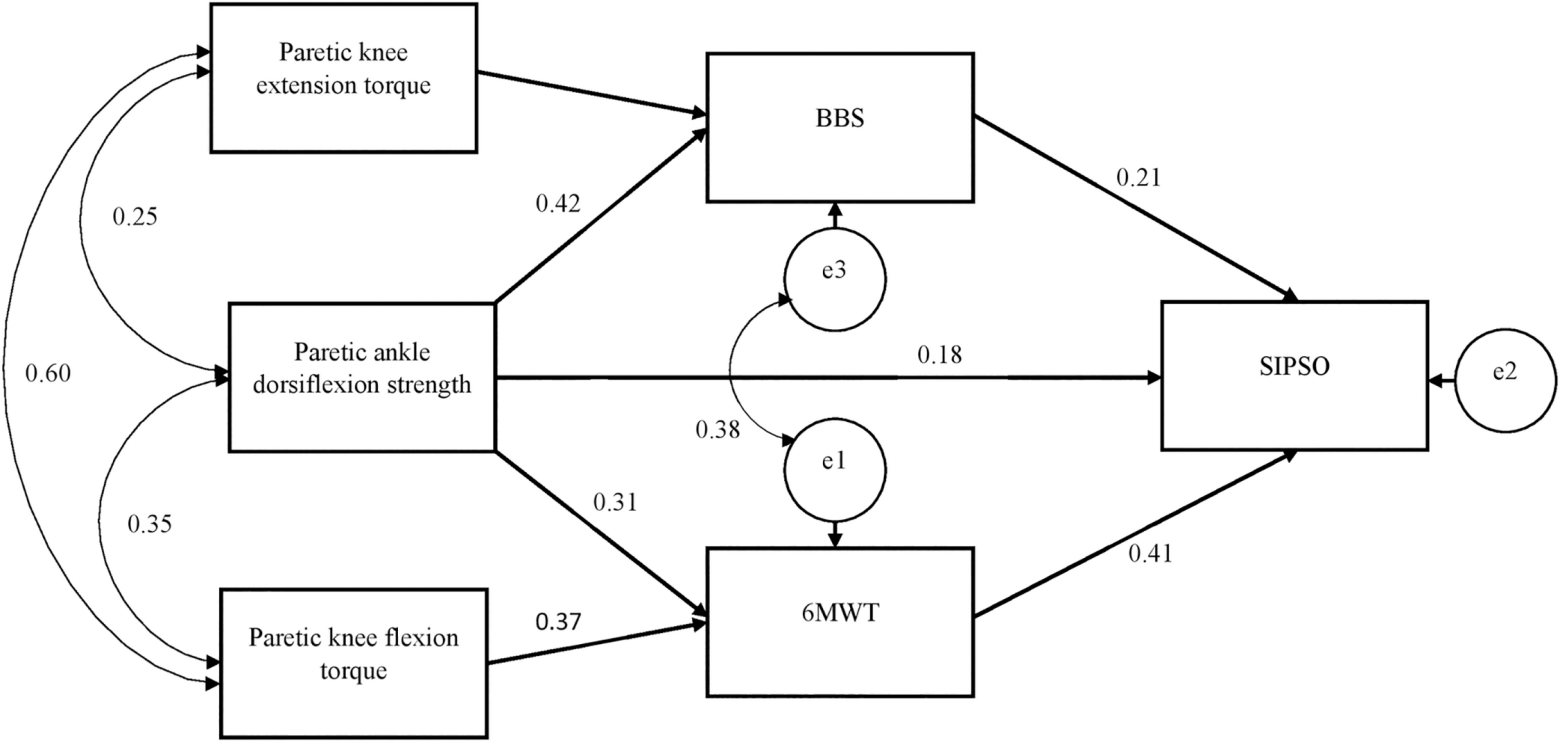
Path diagram shows the relationship between paretic lower-limb muscle strength, walking endurance, balance performance and level of community integration.

Isokinetic paretic knee flexion torque had an indirect association with the SIPSO score mediated by the 6MWT distance. Isokinetic paretic knee extension torque had an indirect association with the SIPSO score mediated by the BBS score. The relationships between muscle strength, balance performance, walking endurance and level of community integration are illustrated in a path diagram (Fig 1). The regression coefficient shows that the 6MWT distance demonstrated the largest direct association with the SIPSO score (*β* = 0.41, *p* <0.001), with every increase of one standard deviation in the 6MWT distance leading to an increase of 0.41 standard deviations in the SIPSO score. The results also show that the BBS score (*β* = 0.21, *p* = 0.021) and isometric dorsiflexion strength (*β* = 0.18, *p =* 0.044) had significant direct associations with the SIPSO score.

The values shown next to the single-headed arrows and double-headed curved arrows are the estimated standardised regression coefficients and estimated correlation coefficients, respectively. All coefficients are statistically significant (*p* ≤ 0.05). e1, e2 and e3 represent the residual of predictions of 6MWT, SIPSO and BBS, respectively. 6MWT: 6-metre walk test; BBS: Berg Balance Scale; SIPSO: Subjective Index of Physical and Social Outcome.]

Initially, the fit indices indicated that the model was a poor fit to the observed data, with CFI and SRMR values of 0.918 and 0.076, respectively. A modification index value of 14.37 indicated that correlation of the residual terms of BBS and 6MWT could significantly improve the model fit. The model fit became satisfactory after the model modification, with CFI and SRMR values of 0.993 and 0.031, respectively.

## Discussion

To the best of our knowledge, this study is the first to evaluate the relationship between paretic lower-limb muscle strength and the level of community integration in community-dwelling chronic stroke survivors using structural equation modelling. Community integration is the ability to live in a usual setting, interact with different people, such as friends and neighbours, and participate in daily activities [2]. A meta-synthesis of the results of 18 qualitative studies revealed that the primary effects of stroke, such as limited mobility, impaired hand functioning and impaired communication ability, remain the major barriers to community integration in stroke survivors [33]. In this study, we found that isometric paretic ankle dorsiflexion strength and isokinetic paretic knee flexion torque indirectly influenced the SIPSO score, mediated by the BBS score and the 6MWT distance. Moreover, isometric paretic ankle dorsiflexion strength demonstrated a significant direct association with the SIPSO score.

In this study, knee muscle strength was measured with an isokinetic dynamometer. A previous study reported that isokinetic training of the knee extensor and flexor in 15 people with chronic stroke resulted in higher levels of perceived participation, as measured by the participation domain of the stroke impact scale, than in the no-treatment control group 5 months after training [34]. Those results suggest a causal relationship between stroke survivors’ isokinetic knee muscle strength and their level of participation.

In this study, isokinetic peak torque was used to measure knee muscle strength and maximum isometric strength was used to measure ankle muscle strength because some of the participants demonstrated severe ankle plantar flexor spasticity, which made measurement of the isokinetic strength of the ankle muscles difficult due to the limited range of motion in the ankle. The maximum isometric ankle muscle strength was therefore measured instead of the isokinetic peak torque.

### SIPSO questionnaire scores

The median SIPSO questionnaire score (28 ± 10) was higher than the scores of 23.3 to 26 reported in previous studies [19, 35, 36]. Despite using similar inclusion criteria and demographic characteristics of stroke survivors, Kilbride et al. [35] reported a SIPSO questionnaire score of 23.3, which is much lower than the median score in our study. The small sample size of only 22 in that study raises a concern about whether the obtained SIPSO questionnaire score could truly reflect the population mean. Two studies with larger sample sizes (261 [19] and 315 [36] chronic stroke survivors) reported a median SIPSO questionnaire score of 26. Both studies recruited stroke survivors from local health service agencies. The studies also included participants who demonstrated poor community ambulation and low levels of community integration [19, 36].

In addition, stroke survivors who were receiving residential care services were included in the study by Trigg and Wood [19]. A subgroup analysis showed that this group scored significantly lower on the SIPSO questionnaire (16.5 vs. 27, *p* = 0.03) [19]. In contrast, we recruited members of local self-help groups, who were likely to be more socially active.

### Influence of isometric ankle dorsiflexion strength

We found that paretic dorsiflexion strength had a direct positive association with the level of community integration in chronic stroke survivors. This result indicates that paretic ankle dorsiflexion strength could have a strong influence on physical functions other than walking endurance and balance performance. Weak paretic ankle dorsiflexion is common after stroke. Isometric paretic ankle dorsiflexion strength measured with a handheld dynamometer in the paretic leg in 60 chronic stroke survivors was reported to be only 35% of that of age-matched healthy control subjects [37].

Ankle dorsiflexion weakness results in insufficient ankle clearance from the floor during the swing phase and reduces the weight-bearing ability of the paretic leg in the mid-stance phase [38]. Based on a sample of 68 chronic stroke survivors, Lin et al. identified that isometric paretic ankle dorsiflexion strength was the primary predictor of comfortable walking speed (*R*^*2*^ = 0.30) and the temporal asymmetry index, which measured the decreased single-leg support time on the paretic leg (*R*^*2*^ = 0.38) [38]. Shiu et al. [39] also reported a significant correlation between isometric paretic ankle dorsiflexion strength and 360° turning time in chronic stroke survivors (*r* = −0.505). Ankle dorsiflexion strength has been suggested to enhance the foot clearance from the ground during turning, thus shortening the completion time for 360° turning [39].

Based on a sample of 73 community-dwelling chronic stroke survivors, Ng and Hui-Chan identified that isometric paretic ankle dorsiflexion strength was the strongest predictor of the timed up and go performance (*R*^*2*^ = 27.5%) [40], which measures the participants’ level of functional mobility. Four of the five items (items 2 to 5) in the questionnaire were found to be related to ambulatory ability to some extent (item 2: the ability to ambulate at home; items 3 and 4: satisfaction and independence of community ambulation; and item 5: shopping). This may explain why paretic ankle dorsiflexion strength contributes significantly to a higher level of community integration as measured by SIPSO in stroke survivors.

### Influence of BBS and indirect associations mediated by BBS

Balance performance measured with the BBS was found to have a direct positive association with the level of community integration. The BBS measures an individual’s stability in performing functional tasks, such as sit-to-stand or stepping up and down. The results agree with those of a previous study that demonstrated that the BBS score was an independent predictor of the RNLI score in a sample of 63 community-dwelling chronic stroke survivors [5]. It is plausible that good balance performance augments the level of self-efficacy in carrying out daily activities and participating in social life, as demonstrated in a previous study [41]. A fear of falling [42] and balance-related self-efficacy [5] were also found to be independent predictors of community integration in stroke survivors. Both factors were strongly associated with balance performance [43, 44]. If the fear of falling and balance-related self-efficacy measures were not included in the model, the influence of balance performance in predicting the level of community integration might have been magnified.

Paretic ankle dorsiflexion strength was found to have a significant direct association with the balance performance measured by BBS and was indirectly associated with the SIPSO score. In contrast, one study reported that isometric ankle dorsiflexion strength was not significantly correlated with the balance performance measured with the functional reach test (*r* = 0.06) in a sample of 30 stroke survivors [45]. The functional reach test measures an individual’s ability to reach forward while standing, thus testing the limit of the participant’s stability. However, another study found that compensatory movements, such as trunk movements, had a stronger influence on the functional reach test results than that of the actual forward displacement of the centre of pressure in healthy elderly subjects [46]. The BBS assesses the stability of a participant in performing multiple functional movements, such as sit-to-stand, turning, stepping up and down, reaching and standing on one leg. Therefore, BBS can be regarded as a more comprehensive tool for measuring balance performance. It is reasonable to suggest that paretic ankle dorsiflexion strength has a strong influence on balance performance because it plays an important role in maintaining ankle stability [47] and facilitating foot clearance during walking [38].

Isokinetic knee extension peak torque was also found to be a significant predictor of balance performance measured by BBS. This result is consistent with the findings of Gerrits et al. [48] and Kobayashi et al. [49] that isometric paretic knee strength had moderate correlation with the BBS score (*r* = 0.64 to 0.76) in 17 and 10 chronic stroke survivors, respectively. Weiss et al. [50] also reported that 12 weeks of resistance training to strengthen the knee extensor and hip muscles led to significant improvement in the BBS score in seven chronic stroke survivors.

### Influence of 6MWT and indirect associations mediated by the 6MWT

The results of this study are consistent with other reports that walking endurance measured by the distance covered in the 6MWT is a significant predictor of the level of community integration (*R*^2^ = 0.326) as measured by the social participation domain of the stroke impact scale in chronic stroke survivors [51]. It is not surprising that walking endurance has a significant association with the level of community integration. As stated earlier, four of the items in the SIPSO questionnaire assess the participants’ abilities in outdoor ambulation. Satisfactory walking endurance could help a stroke survivor attend social gatherings and visit friends or others (item 9). A randomised control trial on 15 chronic stroke survivors demonstrated that 10 weeks of progressive resistance training of the knee extensors and flexors led to a 10% increase in the 6MWT distance (*p <*0.05). More importantly, the percentage changes in the 6MWT distance and the participation domain of the stroke impact scale were strongly correlated (*r* = 0.74, *p* <0.01) [34]. These results [34] further support our finding that isokinetic paretic knee flexion peak torque has a direct association with the 6MWT distance.

Despite our studies having similar inclusion criteria, the results of this study are not consistent with the findings of Liu et al. [42]. They reported that walking endurance measured by the 6MWT was not a significant predictor of the level of community integration (*β* = 0.083, *p* = 0.469) in a sample of 57 chronic stroke survivors [42]. The differences between the findings of the two studies may be partly explained by the different outcome measures. The community integration measure mainly assesses the subjective feelings of being accommodated in the community, whereas SIPSO evaluates the quality and level of engagement in the activities of daily living and social activities.

Paretic isometric ankle dorsiflexion strength was found to be a significant predictor of walking endurance measured by the 6MWT. This result is consistent with Ng and Hui-Chan’s [15] finding that paretic isometric ankle dorsiflexion strength was the strongest independent predictor of the distance covered in the 6MWT and that it could explain up to 48.8% of the variance (*β* = 0.75, *p* <0.001).

Isokinetic knee flexion peak torque was also found to be a significant predictor of walking endurance measured by 6MWT. This result is consistent with the findings of Flansbjer et al. [11] that the paretic isokinetic knee flexion and extension peak torque were independent predictors of walking endurance measured by 6MWT (*R*^*2*^ = 50% and 49%, respectively; *p* < 0.05) in a sample of 50 chronic stroke survivors. In contrast, non-paretic knee flexion and extension peak torque were not significant predictors of walking endurance. A study of the influence of muscle weakness on the walking load of the weakened muscle revealed that weakness of lower limb muscles, including the tibialis anterior and hamstring, would lead to greater loading on the weakened muscles. The increased muscle loading could result in higher energy costs during walking and thus reduce the level of walking endurance [52]. Although the study indicated that muscle weakness could increase the energy costs during walking, it should be noted that the prediction model was based on the gait patterns of healthy participants [52] and may not be applicable to stroke survivors who walk with a pathological gait.

### Model fitting

The modification index revealed that correlating the residual term of the prediction of the BBS score and the 6MWT distance could improve the model’s fit. The result suggests the possible existence of extraneous variables that have not been specified in the model that influence both the BBS score and the 6MWT distance [53]. The presence of such extraneous variables is plausible because only paretic lower-limb muscle strength was used to predict the BBS score and the 6MWT distance in the current model. Other extraneous variables such as the strength of the non-paretic lower limb and severity of stroke might explain a significant portion of the variances of the BBS score and the 6MWT distance. However, further identification of the predictors of the BBS score and the 6MWT distance in stroke survivors was not within the scope of this study.

### Limitations

This study has several limitations. First, the results may have limited generalisability because the subjects were recruited from local patient self-help groups and most were active members who participated in various social and volunteer activities. Stroke survivors over 80 years of age were not recruited. To improve the generalisability of the findings, relatively isolated and older stroke survivors should be recruited in future studies. Second, isometric ankle muscle strength and isokinetic knee muscle peak torque were not measured in a functional position. Thus, the measures may not adequately represent muscle strength during functional activities. Third, we did not conduct a subgroup analysis for stroke survivors with a low level of community integration. Understanding the barriers and facilitators of community integration in isolated stroke survivors may provide more insights for formulation of rehabilitation programmes to facilitate integration. Fourth, the model only explained 43.1% of the variance of the SIPSO score. Given the multi-dimensional nature of community integration, muscle strength, walking endurance and balance performance are likely to be just some of many factors that influence community integration. Moreover, it is possible that other functional measures apart from walking endurance and balance performance could have a strong influence on the SIPSO score. The significant direct association of paretic isometric ankle dorsiflexion strength may reflect the absence from the model of other functional measures that are also strongly influenced by isometric dorsiflexion strength. Lastly, this was a cross-sectional study, and thus causal relationships could not be established between the variables.

## Conclusions

Paretic ankle dorsiflexion strength, walking endurance and balance performance were found to be predictors of the level of community integration in community-dwelling chronic stroke survivors. Paretic ankle dorsiflexion strength influence the level of community integration indirectly, mediated by walking endurance and balance performance. Although the hypothesised model explained only 43.1% of the variance in the level of community integration, our findings nevertheless have implications for facilitating the community integration of stroke survivors.

## Supporting information

S1 File Data generated by the study. (XLSX)
